# Clinical characteristics and high risk factors of patients with Omicron variant strain infection in Hebei, China

**DOI:** 10.3389/fcimb.2023.1294904

**Published:** 2023-12-01

**Authors:** Lihong Wang, Ting Liu, Hongjuan Yue, Jiaojiao Zhang, Qihong Sheng, Ling Wu, Xiaoyu Wang, Mei Zhang, Jing Wang, Jia Wang, Weifang Yu

**Affiliations:** ^1^ Department of Infectious Diseases, The First Hospital of Hebei Medical University, Shijiazhuang, China; ^2^ Department of Endoscopy Center, The First Hospital of Hebei Medical University, Shijiazhuang, China

**Keywords:** new coronavirus infection, omicron, severe disease, clinical features, prognostic risk factors

## Abstract

**Objective:**

The Omicron variant has a weaker pathogenicity compared to the Delta variant but is highly transmissible and elderly critically ill patients account for the majority. This study has significant implications for guiding clinical personalized treatment and effectively utilizing healthcare resources.

**Methods:**

The study focuses on 157 patients infected with the novel coronavirus Omicron variant, from December, 2022, to February, 2023. The objective is to analyze the baseline data, test results, imaging findings and identify risk factors associated with severe illness.

**Results:**

Among the 157 included patients, there were 55 cases in the non-severe group (all were moderate cases) and 102 cases in the severe group (including severe and critical cases). Infection with the Omicron variant exhibits significant differences between non-severe and severe cases (baseline data, blood routine, coagulation, inflammatory markers, cardiac, liver, kidney functions, Chest CT, VTE score, etc.). A multifactorial logistic regression analysis showed that neutrophil percentage >75%, eosinophil percentage <0.4%, D-dimer >0.55 mg/L, PCT >0.25 ng/mL, LDH >250 U/L, albumin <40 g/L, A/G ratio <1.2, cholinesterase<5100 U/L, uric acid >357 mole/L and blood calcium<2.11 mmol/L were the most likely independent risk factors for severe novel coronavirus infection.

**Conclusion:**

Advanced age, low oxygenation index, elevated neutrophil percentage, decreased eosinophil percentage, elevated PCT, elevated LDH, decreased albumin, decreased A/G ratio, elevated uric acid, decreased blood calcium, and elevated D-dimer are independent prognostic risk factors for non-severe patients progressing to severe illness. These factors should be closely monitored and actively treated to prevent or minimize the occurrence of severe illness.

## Introduction

Coronavirus disease 2019 (COVID-19) is an acute respiratory infectious disease caused by severe acute respiratory syndrome coronavirus 2 (SARS-CoV-2, hereinafter referred to as “new coronavirus”), it is highly contagious and the population is generally susceptible (National Health Commission and State Administration of Traditional Chinese Medicine, 2023). COVID-19 has spread rapidly around the world since 2019, and World Health Organization (WHO) first declared it an international pandemic on March 11, 2020, becoming a public health emergency of international concern (Acuti Martellucci et al., 2020). According to data from the Chinese Center for Disease Control and Prevention, as of January 8, 2023, a total of 10,085,921 COVID-19 patients and 33,239 deaths have been diagnosed in our country (including Hong Kong, Macao and Taiwan). According to the World Health Organization (https://covid19.who.int) as of May 31, 2023, the number of confirmed cases of COVID-19 worldwide exceeded 750 million, and the number of deaths exceeded 6.94 million. Multiple organ involvement is a common cause of disease and death, posing a great threat to human life and health (Huang et al., 2020).

The gene of new coronavirus frequently mutates during the epidemic and transmission of the population, and some mutations or recombination will affect the biological characteristics of the virus. As of the end of 2022, WHO has proposed a total of 5 variants of concern (VOC), namely Alpha (B.1.1.7), Beta (B.1.351), Gamma (P.1), Delta (B.1.617.2) and Omicron (B.1.1.529) (Aleem et al., 2023). The Omicron variant strain emerged in the population since November 2021, rapidly spread in China by the end of 2022. The high transmissibility of the novel coronavirus Omicron variant and the huge population base in China have led to a sharp increase in the number of hospitalizations and deaths. Compared with other VOC variants such as Delta, its infectivity, transmissibility, and immune escape ability has been significantly enhanced (Ahmad et al., 2022). According to a briefing by the WHO on December 23, 2021, the household secondary attack rate of the Omicron variant was 15.8%, higher than the 10.3% of the Delta variant, it rapidly replaced the Delta variant as the globally dominant strain in early 2022 (National Health Commission and State Administration of Traditional Chinese Medicine, 2023). Meanwhile, studies have shown that compared to the Beta and Delta variants, the Omicron variant is associated with significantly lower hospitalization and intensive care unit admission rates (da Silva et al., 2022), indicating a lower clinical severity (Yang et al., 2022). Previous reports have mostly focused on the Delta variant. Previous studies have shown that patients with cardiovascular disease or increased cardiovascular risk, diabetes and hypertension are more likely to develop serious forms of COVID-19, or even die, when infected with Delta variant (Peng et al., 2021; Sen et al., 2021; Warren-Gash et al., 2023). In comparison to the Omicron variant, Delta variant infections have been associated with higher levels of LDH, D-dimer, IL-6, and CPR, as these parameters are related to more severe disease (Cao et al., 2020), consistent with the fact that Delta causes more severe illness than Omicron.

Since December 2022, with gradual relaxation policies, the number of individuals testing positive for the novel coronavirus has rapidly increased in China. The number of severe and critical cases has also increased significantly, putting a tremendous strain on the country’s healthcare resources. Particularly in the elderly population, the severity and mortality rates are higher than in the general population. The current phase of the COVID-19 infection poses a significant threat to the health and lives of elderly individuals in China (Viana et al., 2022; Cheng et al., 2023). Although the number of new COVID-19 infections in China has remained low since January 22, 2023, there has been a gradual increase in cases since late April 2023, as reported on the official website of the Chinese Center for Disease Control and Prevention. Furthermore, effective genomic sequences of local COVID-19 cases still correspond to the Omicron variant, indicating that the novel coronavirus (Omicron variant) will likely coexist with humans in the long term and continue to pose a threat to the health and lives of elderly patients (Wang et al., 2023). To gain a deeper understanding of the clinical characteristics of patients infected with the Omicron variant, this study retrospectively analyzed relevant cases admitted to our hospital from December 1, 2022, to February 12, 2023. All patients were categorized into non-severe and severe groups, aiming to identify factors associated with severe infection. This research seeks to provide reference criteria for the clinical diagnosis and treatment of COVID-19 patients, particularly in reducing the severity and in-hospital mortality rates among elderly patients.

## Methods

### Study design and participants

**Figure 1:**
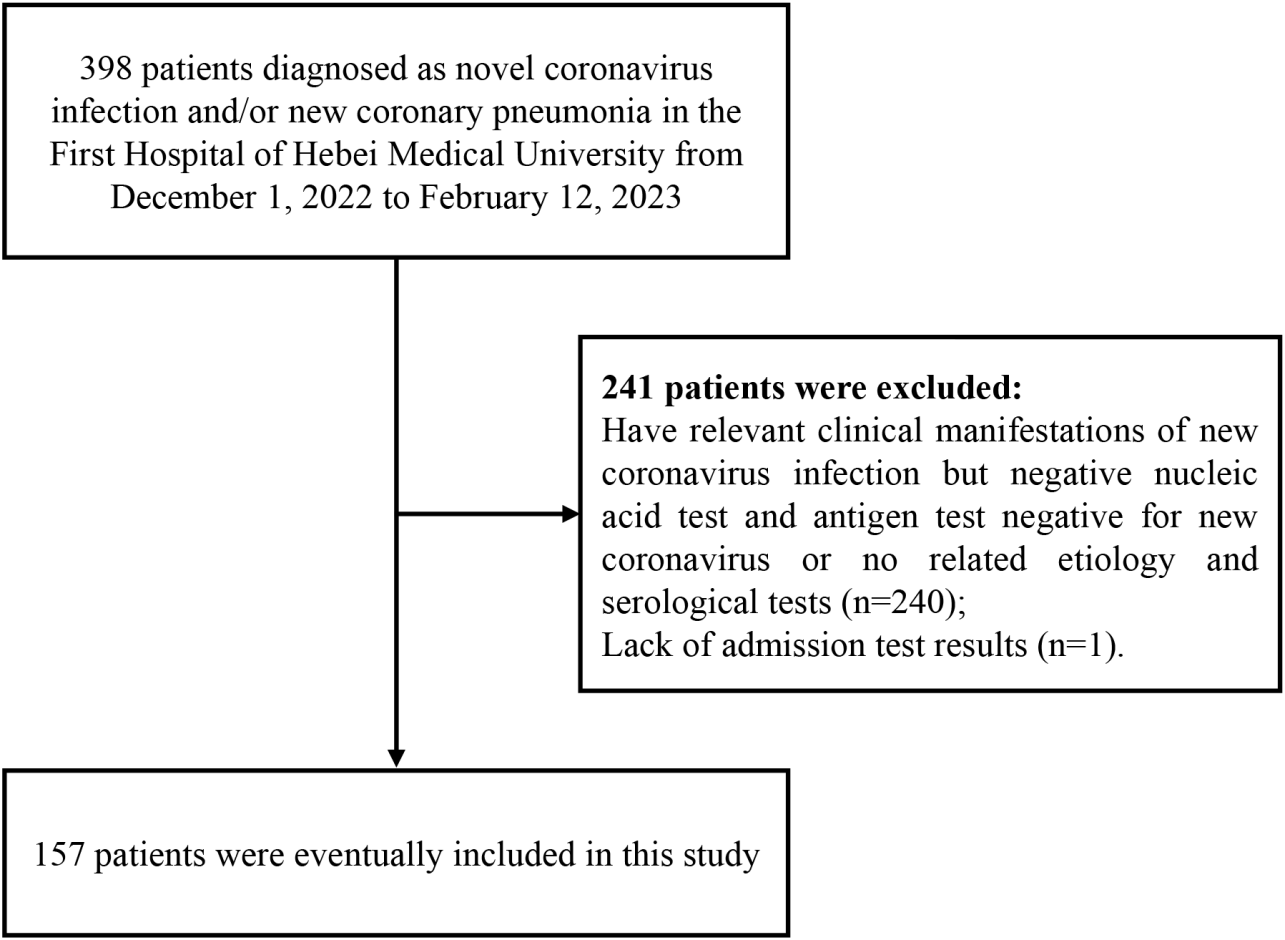
Recruitment flowchart of patients with Omicron variant strain infection.

In this retrospective study, we included patients diagnosed with novel coronavirus infection and admitted to the First Hospital of Hebei Medical University from December 1, 2022, to February 12, 2023. According to the monitoring data from the Chinese Center for Disease Control and Prevention, the Omicron variant has been dominant among the patients infected with the novel coronavirus since December 2022. The inclusion criteria were as follows: for patients admitted before January 8, 2023, we followed the screening criteria outlined in the “Diagnosis and Treatment Protocol for Novel Coronavirus Pneumonia (Trial 9th Edition)” issued by the National Health Commission of China and included all confirmed cases; for patients admitted between January 8 and February 12, 2023, we followed the screening criteria in the “Diagnosis and Treatment Protocol for Novel Coronavirus Infection (Trial 10th Edition)” also issued by the National Health Commission of China. Exclusion criteria included patients with clinical symptoms related to COVID-19 but tested negative for novel coronavirus nucleic acid or antigen, or those for whom relevant etiological or serological examinations were not conducted. Additionally, one patient who lacked admission laboratory results was excluded from the study (**Figure 1**: Flowchart depicting the inclusion and exclusion process of the 398 patients’ clinical data obtained). Therefore, 157 patients were ultimately included in the analysis. According to the “Diagnosis and Treatment Protocol for Novel Coronavirus Infection (Trial 10th Edition)” issued by the National Health Commission of China, patients were classified into four groups (mild, moderate, severe, and critical). In order to better understand their clinical characteristics and provide guidance for early diagnosis and timely treatment of severe COVID-19 patients, we divided all patients into two groups: the non-severe group (including moderate cases) and the severe group (including severe and critical cases). This study has been approved by the Ethics Committee of the First Hospital of Hebei Medical University (Ethics Approval No.: S00138).

### Data collection

The patients’ medical records were collected and analyzed by the research team in the Department of Infectious Diseases at the First Hospital of Hebei Medical University. The following information was obtained from the hospital’s electronic medical record system: demographic data, medical history, vital signs on admission, routine blood tests, imaging results, etc. The medical records of the patients were independently reviewed by three researchers (J.W., L.W., H.Y.) to ensure data accuracy. All laboratory tests and imaging procedures were conducted by the standardized and standardized processes in the laboratory, imaging department, ultrasound department, and other relevant departments of the First Hospital of Hebei Medical University.

### Statistical analysis

For continuous variables, a Shapiro-Wilk test was performed to assess the normality of each variable. Normally distributed continuous variables are presented as mean ± standard deviation (SD) and analyzed using *t*-tests. Non-normally distributed continuous variables are presented as median and interquartile range, and analyzed using the Wilcoxon rank-sum test. Categorical variables are presented as counts and percentages, and differences between the non-severe and severe groups are compared using the chi-square test or Fisher’s exact test. Univariate and multivariate logistic regression models were used to identify potential independent risk factors associated with severe COVID-19. In our study, all covariates in the univariate logistic regression were binary categorical data, including demographic data, medical history, vital signs on admission, and laboratory tests. Due to the limited sample size (n=157) and missing data for some laboratory parameters, only statistically significant risk factors identified in the univariate logistic regression analysis were included in the final multivariate model. Odds ratios (ORs) and 95% confidence intervals (95% CIs) were calculated for both univariate and multivariate regression analyses. Additionally, not all patients included in the study underwent all laboratory tests and examinations, leading to inconsistent sample sizes for some variables compared to the total number of cases. All statistical analyses were conducted using IBM SPSS Statistics software (version 25), and a significance level of 0.05 was used.

## Results

### Demographic and clinical characteristics

According to data from the Hebei Provincial Center for Disease Control and Prevention, this study included 157 hospitalized patients diagnosed with Omicron variant infection (**Table 1**). The median age was 73 years (IQR: 62-83; range: 17-96 years), and 100 (63.7%) were male. The study population comprised 55 (35.0%) moderate cases (non-severe group) and 102 (65.0%) severe and critical cases (severe group). Among the 157 patients included in the study, the majority were aged 60 years and above (n=123, 78.3%), and the proportion of elderly patients was significantly higher in the severe group compared to the non-severe group (*P*=0.013). The median BMI was 23.9 kg/m^2 (IQR: 21.2-26.7), with 68 (48.2%) patients having a BMI ≥24, indicating overweight status, and the proportion of overweight individuals was higher in the severe group compared to the non-overweight group (overweight: n=46, 52.9%; non-overweight: n=41, 47.1%). The smoking rate was significantly higher in the severe group compared to the non-severe group (*P*=0.015). Hypertension, diabetes, coronary heart disease, and a history of surgery were common comorbidities, a small number of patients had concomitant chronic obstructive pulmonary disease, asthma, cerebral infarction, renal failure, organ failure, a history of cancer, and immunosuppression. The occurrence of previous surgeries was significantly higher in the severe group compared to the non-severe group (*P*=0.005).

**Table 1:** Demographic and clinical characteristics of non-severe and severe groups.

	All patients (n=157)	Non-severe (n=55)	Severe (n=102)	*P* value
Ages, years	73(62,83)	70(53,78)	77(68,83)	0.001
<60	34(21.7%)	18(32.7%)	16(15.7%)	0.013
≥60	123(78.3%)	37(67.3%)	86(84.3%)	
Sex				0.292
Men	100(63.7%)	32(58.2%)	68(66.7%)	
Women	57(36.3%)	23(41.8%)	34(33.3%)	
BMI, kg/m^2	23.9(21.2,26.7)	23.1(20.7,25.8)	24.4(21.9,26.7)	0.202
<24	73/141(51.8%)	32/54(59.3%)	41/87(47.1%)	0.161
≥24	68/141(48.2%)	22/54(40.7%)	46/87(52.9%)	
Smoking	30/151(19.9%)	5/54(9.3%)	25/97(25.8%)	0.015
Drinking	22/151(14.6%)	6/54(11.1%)	16/97(16.5%)	0.369
Any comorbidity				
Hypertension	86/155(55.5%)	26/54(48.1%)	60/101(59.4%)	0.179
Diabetes	46/152(30.3%)	16/53(30.2%)	30/99(30.3%)	0.988
Chronic obstructive pulmonary disease	8(5.1%)	3(5.5%)	5(4.9%)	0.881
Asthma	4(2.5%)	1(1.8%)	3(2.9%)	1.000
Coronary heart disease	42(26.8%)	15(27.3%)	27(26.5%)	0.914
Cerebral infarction	27(17.2%)	8(14.5%)	19(18.6%)	0.518
Renal failure	5(3.2%)	0(0.0%)	5(4.9%)	0.233
Organ failure	7(4.5%)	0(0.0%)	7(6.9%)	0.114
Surgery	54(34.4%)	11(20.0%)	43(42.2%)	0.005
Tumour	19(12.1%)	5(9.1%)	14(13.7%)	0.396
Immunosupression	17(10.8%)	7(12.7%)	10(9.8%)	0.574
Vital sign				
Temperature, °C	36.7(36.5,37.2)	36.6(36.3,37.3)	36.7(36.5,37.2)	0.681
≤37.3	82/155(52.9%)	29/55(52.7%)	53/100(53.0%)	0.974
>37.3	73/155(47.1%)	26/55(47.3%)	47/100(47.0%)	
Respiratory rate, breathes per min	20.0(18.0,20.0)	20.0(18.0,20.0)	20.0(18.0,22.0)	0.616
≤24	119/155(76.8%)	47/55(85.5%)	72/100(72.0%)	0.058
>24	36/155(23.2%)	8/55(14.5%)	28/100(28.0%)	
Heart rate	84.0(78.0,95.0)	84.0(79.0,94.0)	86.5(77.3,96.0)	0.887
≤100	136/155(87.7%)	50/55(90.9%)	86/100(86.0%)	0.373
>100	19/155(12.3%)	5/55(9.1%)	14/100(14.0%)	
Systolic pressure, mmHg	135.9±21.8	130.2±18.8	139.0±22.8	0.015
≤140	93/155(60.0%)	41/55(74.5%)	52/100(52.0%)	0.006
>140	62/155(40.0%)	14/55(25.5%)	48/100(48.0%)	
Diastolic pressure, mmHg	79.1±12.9	82.1±10.6	77.4±13.7	0.019
≤90	127/155(81.9%)	44/55(80.0%)	83/100(83.0%)	0.642
>90	28/155(18.1%)	11/55(20.0%)	17/100(17.0%)	
Oxygenation index (PO2/FiO2)	340.5(271.0,406.1)	398.3(362.5,497.6)	316.7(264.3,385.7)	<0.001

Among the 155 patients, 73 (47.1%) had fever, 36 (23.3%) had increased respiratory rate, and 19 (12.3%) had increased heart rate. Among the 155 patients, only 62 (40.0%) had elevated systolic blood pressure, 28 (20.0%) had elevated diastolic blood pressure, and the proportion of patients with elevated systolic blood pressure was significantly higher in the severe group compared to the non-severe group (*P*=0.006). The median oxygenation index for 129 patients was 340.5 (IQR: 271.0-406.1), 398.3 (IQR: 362.5-497.6) for non-severe group patients, and 316.7 (IQR: 264.3-385.7) for severe group patients; and the oxygenation index of severe group patients was significantly lower than that of non-severe group (*P*<0.001).

### The blood routine, coagulation function, and inflammatory marker tests

As shown in **Table 2**, there are significant differences between the non-severe and severe groups in blood routine, coagulation function, and inflammatory marker test results. The median white blood cell count was significantly higher in the severe group compared to the non-severe group (*P*=0.001). The proportion of patients with elevated white blood cell count was significantly higher in the severe group compared to the non-severe group (*P*=0.003). Neutrophil percentage was elevated in 85 patients, and the proportion of patients with elevated neutrophil percentage was significantly higher in the severe group compared to the non-severe group (*P*<0.001). The majority of patients showed significant decreases in lymphocyte percentage (n=107, 68.2%) and eosinophil percentage (n=95, 61.7%), and the proportion of patients with decreased lymphocyte percentage and eosinophil percentage was significantly higher in the severe group compared to the non-severe group (*P*<0.001). The median NLR and median PLR levels in the severe group were significantly higher than those in the non-severe group (*P*<0.001).

**Table 2:** Laboratory results of blood routine, coagulation and inflammation indicators in non-severe and severe groups.

	All patients (n=157)	Non-severe (n=55)	Severe (n=102)	*P* value
Blood routine tests				
White blood cell count, × 10^9^ per L	5.9(4.3,8.2)	4.7(4.0,6.6)	6.4(4.6,9.6)	0.001
<3.5	15(9.6%)	6(10.9%)	9(8.8%)	0.003
3.5-9.5	114(72.6%)	47(85.5%)	67(65.7%)	
>9.5	28(17.8%)	2(3.6%)	26(25.5%)	
Neutrophil percentage,%	77.3(66.4,88.0)	67.0(59.9,74.1)	83.3(73.1,90.5)	<0.001
<40%	2(1.3%)	0(0.0%)	2(2.0%)	<0.001
40%-75%	70(44.6%)	44(80.0%)	26(25.5%)	
>75%	85(54.1%)	11(20.0%)	74(72.5%)	
Lymphocyte percentage, %	12.8(6.3,22.6)	21.1(13.4,28.6)	9.9(4.8,16.2)	<0.001
<20%	107(68.2%)	24(43.6%)	83(81.4%)	<0.001
20%-50%	48(30.6%)	31(56.4%)	17(16.7%)	
>50%	2(1.3%)	0(0.0%)	2(2.0%)	
Eosinophil percentage, %	0.2(0.0,0.8)	0.5(0.2,1.4)	0.1(0.0,0.4)	<0.001
<0.4%	95/154(61.7%)	20/53(37.7%)	75/101(74.3%)	<0.001
≥0.4%	59/154(38.3%)	33/53(62.3%)	26/101(25.7%)	
Neutrophil to lymphocyte ratio	6.2(3.1,13.8)	3.1(2.1,5.3)	8.4(4.9,19.2)	<0.001
Platelet to lymphocyte ratio	240.0(154.1,371.0)	171.0(120.2,255.9)	281.4(186.9,474.4)	<0.001
Haemoglobin, g/L	120.6±19.5	124.5±17.4	118.5±20.3	0.066
<130	109/157(69.4%)	35/55(63.6%)	74/102(72.5%)	0.248
≥130	48/157(30.6%)	20/55(36.4%)	28/102(27.5%)	
Coagulation tests				
Prothrombin time, s	11.5(10.7,12.7)	11.3(10.9,12.5)	11.6(10.6,13.0)	0.633
≤12.5	110/154(71.4%)	40/53(75.5%)	70/101(69.3%)	0.421
>12.5	44/154(28.6%)	13/53(24.5%)	31/101(30.7%)	
International normalized ratio	1.03(0.97,1.03)	1.02(0.98,1.11)	1.04(0.96,1.16)	0.561
≤1.4	147/153(96.1%)	53/53(100.0%)	94/100(94.0%)	0.167
>1.4	6/153(3.9%)	0/53(0%)	6/100(6.0%)	
D-dimer, mg/L	0.9(0.5,2.3)	0.5(0.3,0.9)	1.4(0.7,4.7)	<0.001
≤0.55	42/153(28.1%)	28/52(53.8%)	15/101(14.9%)	<0.001
>0.55	110/153(71.9%)	24/52(46.2%)	86/101(71.9%)	
Fibrinogen, g/L	4.7(4.0,5.4)	4.2(3.6,5.2)	4.8(4.1,5.5)	0.006
≤4.98	93/151(61.6%)	38/52(73.1%)	55/99(55.6%)	0.035
>4.98	58/151(38.4%)	14/52(26.9%)	44/99(44.4%)	
Fibrin degradation products	2.8(1.8,6.2)	1.8(0.7,1.9)	4.0(2.5,7.4)	<0.001
≤5	38/54(70.4%)	12/12(100.0%)	26/42(61.9%)	0.028
>5	16/54(29.6%)	0/12(0.0%)	16/42(38.1%)	
Inflammatory indicators				
Interleukin-1β, pg/mL	1.5(1.1,2.4)	1.4(0.6,1.7)	1.7(1.2,3.0)	0.025
≤12.4	82/88(93.2%)	24/25(96.0%)	58/63(92.1%)	0.848
>12.4	6/88(6.8%)	1/25(4.0%)	5/63(7.9%)	
Interleukin-2, pg/mL	1.4(0.8,2.6)	1.3(0.6,1.7)	1.6(0.9,2.8)	0.101
≤5.71	81/88(92.0%)	24/25(96.0%)	57/63(90.5%)	0.669
>5.71	7/88(8.0%)	1/25(4.0%)	6/63(9.5%)	
Interleukin-4, pg/mL	1.3(0.6,2.4)	1.0(0.4,1.6)	1.4(0.7,2.6)	0.084
≤3	75/88(85.2%)	22/25(88.0%)	53/63(84.1%)	0.898
>3	13/88(14.8%)	3/25(12.0%)	10/63(15.9%)	
Interleukin-5, pg/mL	1.5(0.9,2.0)	1.3(0.8,1.9)	1.5(0.9,2.1)	0.511
≤3.1	75/88(85.2%)	23/25(92.0%)	52/63(82.5%)	0.427
>3.1	13/88(14.8%)	2/25(8.0%)	11/63(17.5%)	
Interleukin-6, pg/mL	23.6(5.1,84.3)	4.7(2.0,31.4)	35.6(8.8,107.9)	<0.001
≤7	40/133(30.1%)	22/39(56.4%)	18/94(19.1%)	<0.001
>7	93/133(69.9%)	17/39(46.3%)	76/94(80.9%)	
Interleukin-8, pg/mL	33.5(15.5,69.3)	17.1(8.9,41.6)	46.1(21.1,81.3)	0.002
≤20.6	31/88(35.2%)	16/25(64.0%)	15/63(23.8%)	<0.001
>20.6	57/88(64.8%)	9/25(36.0%)	48/63(76.2%)	
Interleukin-10, pg/mL	5.2(2.9,8.3)	2.5(1.7,5.3)	6.2(4.1,9.0)	<0.001
≤4.91	40/88(45.5%)	17/25(68.0%)	23/63(36.5%)	0.007
>4.91	48/88(54.5%)	8/25(32.0%)	40/63(63.5%)	
Interleukin-12, pg/mL	1.4(0.8,2.4)	1.3(0.6,1.6)	1.6(0.8,2.6)	0.074
≤3.4	78/88(88.6%)	25/25(100.0%)	53/63(84.1%)	0.081
>3.4	10/88(11.4%)	0/25(0.0%)	10/63(15.9%)	
Interferon-γ, pg/mL	2.4(1.6,3.8)	2.0(1.0,3.5)	2.7(1.7,4.6)	0.057
≤7.42	79/88(89.8%)	24/25(96.0%)	55/63(87.3%)	0.410
>7.42	9/88(10.2%)	1/25(4.0%)	8/63(12.7%)	
Interferon-α, pg/mL	2.9(1.3,6.6)	2.2(1.0,6.4)	3.1(1.4,8.5)	0.238
≤8.5	70/88(79.5%)	23/25(92.0%)	47/63(74.6%)	0.068
>8.5	18/88(20.5%)	2/25(8.0%)	16/63(25.4%)	
Tumor necrosis factor-α, pg/mL	1.6(0.9,2.8)	1.58(1.03,2.44)	1.57(0.92,2.96)	0.868
≤4.6	75/88(85.2%)	22/25(88.0%)	53/63(84.1%)	0.898
>4.6	13/88(14.8%)	3/25(12.0%)	10/63(14.9%)	
Erythrocyte sedimentation rate, mm/h	42.0(25.5,64.0)	28.0(15.0,52.0)	53.5(34.8,70.8)	0.006
≤15	10/69(14.5%)	7/29(24.1%)	3/40(7.5%)	0.112
>15	59/69(85.5%)	22/29(75.9%)	37/40(92.5%)	
C-rective protein, mg/L	51.2(23.5,92.6)	29.4(9.9,57.9)	64.6(36.0,107.4)	<0.001
≤77	97/145(66.9%)	44/52(84.6%)	53/93(57.0%)	0.001
>77	48/145(33.1%)	8/52(15.4%)	40/93(43.0%)	
Procalcitonin, ng/mL	0.13(0.07,0.50)	0.06(0.03,0.11)	0.24(0.09,1.11)	<0.001
<0.1	48/128(37.4%)	23/36(63.9%)	25/92(27.2%)	<0.001
≥0.1 to <0.25	32/128(25.0%)	11/36(30.6%)	21/92(22.8%)	
≥0.25 to <0.5	16/128(12.5%)	0/36(0.0%)	16/92(17.4%)	
≥0.5	32/128(25.0%)	2/36(5.6%)	30/92(32.6%)	

There were no significant differences between the severe and non-severe groups in terms of median prothrombin time (PT) and median INR. Additionally, the proportions of patients with elevated D-dimer, fibrinogen, and fibrinogen degradation product levels were significantly higher in the severe group compared to the non-severe group (D-dimer: *P*<0.001; fibrinogen: *P*=0.035; fibrinogen degradation product: *P*=0.028).

A total of 88 patients underwent a 12-cytokine panel examination, with 25 patients in the non-severe group and 63 patients in the severe group. The proportions of patients with elevated levels of IL-1β, IL-2, IL-4, IL-5, IL-12, IFN-γ, IFN-α, and TNF-α were higher in the severe group than in the non-severe group, but there were no statistically significant differences. There was a significantly higher proportion of patients with elevated IL-6, IL-8 and IL-10 levels in the severe group compared to the non-severe group (IL-6, IL-8: *P*<0.001; IL-10: *P*=0.007). In addition, 69 patients underwent erythrocyte sedimentation rate (ESR) testing, there was no statistically difference between the proportion of patients with ESR values above 15 mm/h in the severe group and the non-severe group. There was a significantly higher proportion of patients with elevated C-reactive protein (CRP) and PCT levels in the severe group compared to the non-severe group (CRP: *P*=0.001; PCT: *P*<0.001).

### Cardiac, liver, and renal function tests

According to **Table 3**, the proportion of patients with elevated levels of cardiac troponin I (catnip), creatine kinase (CK), creatine kinase-MB (CK-MB), and alpha-hydroxybutyrate dehydrogenase (α-HBDH) in the severe group was significantly higher than in the non-severe group (catnip, CK, α-HBDH: *P*<0.001; CK-MB: *P*=0.001). Most patients showed elevated levels of B-type natriuretic peptide (BNP) and lactate dehydrogenase (LDH) (BNP: n=60, 52.6%; LDH: n=81, 52.9%), and the proportion of patients with elevated BNP and LDH levels in the severe group was significantly higher than in the non-severe group (*P*<0.001).

**Table 3:** Laboratory tests of heart function, liver function and kidney function in severe and non-severe groups.

	All patients (n=157)	Non-severe (n=55)	Severe (n=102)	*P* value
Heart function tests				
Troponin I, ng/mL	0.016(0.007,0.050)	0.008(0.004,0.015)	0.024(0.009,0.077)	<0.001
≤0.023	74/122(60.7%)	31/35(88.6%)	43/87(49.4%)	<0.001
>0.023	48/122(39.3%)	4/35(11.4%)	44/87(50.6%)	
BNP, pg/mL	124.0(26.8,326.8)	32.0(14.8,140.5)	186.5(58.5,421.8)	<0.001
≤100	54/114(47.4%)	28/38(78.7%)	26/76(34.2%)	<0.001
>100	60/114(52.6%)	10/38(26.3%)	50/76(65.8%)	
Creatine kinase, U/L	81.5(43.8,148.8)	64.5(43.5,97.5)	106.0(43.5,288.5)	0.002
≤200	125/154(81.2%)	52/52(100.0%)	73/102(71.6%)	<0.001
>200	29/154(18.8%)	0/52(0.0%)	29/102(28.4%)	
Creatine kinase-MB, ng/mL	3.2(2.4,4.6)	2.8(2.1,3.5)	3.6(2.4,5.3)	0.001
≤5.0	123/153(80.4%)	49/51(96.1%)	74/102(72.5%)	0.001
>5.0	30/153(19.6%)	2/51(3.9%)	28/102(27.5%)	
Lactate dehydrogenase, U/L	265.0(200.0,392.0)	200.0(180.0,250.0)	299.5(228.5,443.8)	<0.001
≤250	72/153(47.1%)	39/51(76.5%)	33/102(32.4%)	<0.001
>250	81/153(52.9%)	12/51(23.5%)	69/102(67.6%)	
α-Hydroxybutyrate dehydrogenase, U/L	163.0(133.0,243.0)	134.0(113.0,156.0)	197.5(146.0,293.3)	<0.001
≤182	90/153(58.8%)	43/51(84.3%)	47/102(46.1%)	<0.001
>182	63/153(41.2%)	8/51(15.7%)	55/102(53.9%)	
Liver function tests				
AST, U/L	29.2(21.5,44.4)	23.5(17.4,36.3)	32.7(24.3,53.6)	<0.001
≤35	101/155(65.2%)	38/53(71.7%)	63/102(61.8%)	0.218
>35	54/155(34.8%)	15/53(28.3%)	39/102(38.2%)	
ALT, U/L	22.6(14.3,39.7)	25.7(14.2,41.0)	22.3(39.9,63.3)	0.876
≤40	117/155(75.5%)	40/53(75.5%)	77/102(75.5%)	0.998
>40	38/155(24.5%)	13/53(24.5%)	25/102(24.5%)	
Albumin, g/L	33.0±4.9	35.8±4.9	31.5±4.2	<0.001
<40	143/155(92.3%)	43/53(81.1%)	100/102(98.0%)	<0.001
40-55	12/155(7.7%)	10/53(18.9%)	2/102(2.0%)	
A/G	1.3±0.3	1.5±0.3	1.2±0.3	<0.001
<1.20	63/155(40.6%)	12/53(22.6%)	51/102(50.0%)	<0.001
≥1.20	92/155(59.4%)	41/53(77.4%)	51/102(50.0%)	
Cholinesterase, U/L	4953.9±1890.0	5820.4±2012.6	4503.8±1662.1	<0.001
<5100	90/155(98.1%)	21/53(39.6%)	69/102(67.6%)	0.001
≥5100	65/155(41.9%)	32/53(60.4%)	33/102(32.4%)	
Blood lipids				
Total cholesterol, mmol/L	3.7(3.1,4.4)	3.9(3.0,4.9)	3.5(3.1,4.2)	0.178
<2.8	20/122(16.4%)	5/43(11.5%)	15/79(19.0%)	0.556
2.8-3.7	42/122(34.4%)	15/43(34.9%)	27/79(34.2%)	
>3.7	60/122(49.2%)	23/43(53.5%)	37/79(46.8%)	
High density lipoprotein cholesterol, mmol/L	0.9(0.8,1.1)	0.9(0.8,1.1)	0.9(0.7,1.2)	0.488
<1.03	76/122(62.3%)	28/43(65.1%)	48/79(60.8%)	0.845
1.03-1.55	42/122(34.4%)	14/43(32.6%)	28/79(35.4%)	
>1.55	4/122(3.3%)	1/43(2.3%)	3/79(3.8%)	
Low density lipoprotein cholesterol, mmol/L	2.3±0.7	2.4±0.7	2.3±0.6	0.209
<3.37	111/122(91.0%)	38/43(88.4%)	73/79(92.4%)	0.457
≥3.37	11/122(9.0%)	5/43(11.6%)	6/79(7.6%)	
Kidney function tests				
Creatinine, μmol/L	67.9(57.2,96.1)	63.1(53.9,85.3)	71.0(57.9,106.0)	0.042
≤73	90/156(57.7%)	35/54(64.8%)	55/102(53.9%)	0.190
>73	66/156(42.3%)	19/54(35.2%)	47/102(46.1%)	
Uric acid, μmol/L	215.4(156.9,298.1)	218.5(169.0,274.5)	209.8(149.0,327.6)	0.877
≤357	132/155(85.2%)	51/53(96.2%)	81/102(79.4%)	0.005
>357	23/155(14.8%)	2/53(3.8%)	21/102(20.6%)	
Sodium, mmol/L	138.0(134.8,140.2)	138.0(135.0,140.0)	137.0(134.0,141.0)	0.341
<137.0	63/154(40.9%)	15/53(28.3%)	48/101(47.5%)	0.021
≥137.0	91/154(59.1%)	38/53(71.7%)	53/101(52.5%)	
Potassium, mmol/L	3.9(3.6,4.3)	3.91(3.69,4.24)	3.93(3.54,4.49)	0.854
<3.5	32/154(20.8%)	10/53(18.9%)	22/101(21.8%)	0.672
≥3.5	122/154(79.2%)	43/53(81.1%)	79/101(78.2%)	
Calcium, mmol/L	2.1(1.9,2.2)	2.2(2.1,2.3)	2.0(1.9,2.1)	<0.001
<2.11	88/152(57.9%)	11/53(20.8%)	77/99(77.8%)	<0.001
≥2.11	64/152(42.1%)	42/53(79.2%)	22/99(22.2%)	
Lymphocyte subpopulation				
CD4+T lymphocyte percentage, %	40.9(35.4,47.5)	42.8(36.5,48.0)	40.7(31.8,47.6)	0.317
CD4+/CD8+	1.8(1.2,2.4)	1.9(1.3,2.4)	1.7(1.2,2.4)	0.775

AST and ALT levels showed no statistical difference between the non-severe and severe groups. Most patients exhibited decreased levels of albumin and cholinesterase, and the proportion of patients with decreased albumin, albumin/globulin (A/G) ratio, and cholinesterase levels in the severe group was significantly higher than in the non-severe group (albumin: *P*<0.001; A/G ratio, cholinesterase: *P*=0.001). Total cholesterol, high-density lipoprotein cholesterol (HDL-C), and low-density lipoprotein cholesterol (LDL-C) levels did not show statistical differences between the non-severe and severe groups.

In the severe group, there was a higher proportion of patients with elevated levels of creatinine, uric acid, decreased sodium, decreased potassium, and decreased calcium compared to the non-severe group (elevated creatinine: *P*=0.190; elevated uric acid: *P*=0.005; decreased sodium: *P*=0.021; decreased potassium: P=0.672; decreased calcium: *P*<0.001). The median percentage of CD4+ T lymphocytes and the median CD4+/CD8+ ratio in the severe group were lower than in the non-severe group, but without statistical significance.

### Radiological and other examination findings

**Figure 2:**
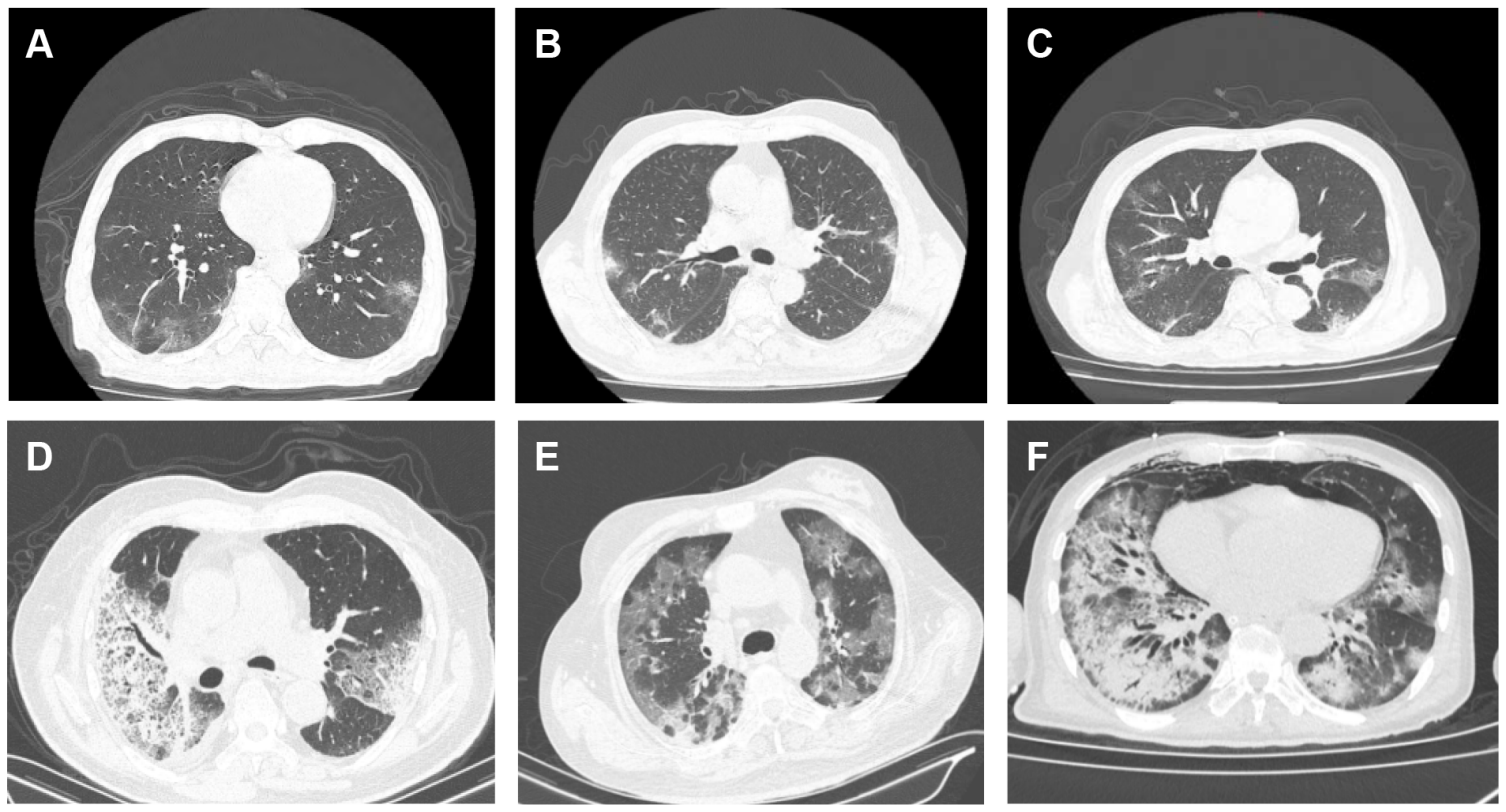
Chest CT images of patients with infection of the novel coronavirus Omicron variant. **(A–C)** Non-severe cases showing multiple patchy ground-glass opacities and high-density lesions with unclear borders in both lower lobes: **(A)** A 62-year-old male with fever, cough, and sputum for 12 days; **(B)** A 26-year-old female with fever for 6 days; **(C)** An 89-year-old male with fever for 7 days. **(D, E)** Severe cases: **(D)** An 81-year-old male with fever for 7 days, showing multiple patchy high-density lesions in both lungs; **(E)** A 59-year-old female with fever and dyspnea for 7 days, showing multiple patchy high-density lesions and thickened interlobular septa in both lungs; **(F)** A 64-year-old male with fever for 13 days, showing multiple patchy high-density and ground-glass opacities with blurred margins in both lungs.

We have displayed all the test results in **Table 4**. Among the 157 patients, 124 patients completed chest CT scans, of which 81 patients (65.3%) showed patchy and ground-glass opacities, 38 patients (30.6%) showed patchy and ground-glass opacities with consolidation, and only 5 patients (4.0%) showed consolidation alone. There was a statistically significant difference in different chest CT manifestations between the two groups (*P*=0.037). The majority of patients in both the non-severe group and the severe group showed patchy and ground-glass opacities (non-severe group: n=33, 70.2%; severe group: n=48, 62.3%), followed by patchy and ground-glass opacities with consolidation (non-severe group: n=10, 21.3%; severe group: n=28, 36.4%). Compared to the severe group, the non-severe group had a higher proportion of patients with pure patchy and ground-glass opacities, while the severe group had a higher proportion of patients with consolidation (**Figure 2**).

**Table 4:** Radiological and other examination results in severe and non-severe groups.

	All patients (n=157)	Non-severe (n=55)	Severe (n=102)	*P* value
Co-infection				<0.001
Not	31/150(20.7%)	11/51(21.6%)	20/99(20.2%)	
Bacterium	69/150(46.0%)	36/51(70.6%)	33/99(33.3%)	
Fungus	19/150(12.7%)	2/51(3.9%)	17/99(17.2%)	
Both	31/150(20.7%)	2/51(3.9%)	29/99(29.3%)	
Pulmonary CT				0.037
Patchy shadow or Ground glass opacity	81/124(65.3%)	33/47(70.2%)	48/77(62.3%)	
Consolidation shadow	5/124(4.0%)	4/47(8.5%)	1/77(1.3%)	
All	38/124(30.6%)	10/47(21.3%)	28/77(36.4%)	
Lower extremity Doppler ultrasound (DUS)	51/137(37.2%)	12/35(34.3%)	39/102(38.25)	0.677
VTE risk score				0.025
0-2	77/153(50.3%)	34/53(64.2%)	43/100(43.0%)	
3-4	75/153(49.0%)	19/53(35.8%)	56/100(56.0%)	
≥5	1/153(0.7%)	0/53(0.0%)	1/100(1.0%)	
Abdominal ultrasonography (US)	67/87(77.0%)	20/28(71.4%)	47/59(79.7%)	0.394
Electrocardiogram (ECG)				0.007
Normal	33/112(29.5%)	23/50(46.0%)	10/62(16.1%)	
Abnormal ST-T segment	23/112(20.5%)	8/50(16.0%)	15/62(24.2%)	
Abnormal impulse conduction	34/112(30.4%)	11/50(22.0%)	23/62(37.1%)	
All	22/112(19.6%)	8/50(16.0%)	14/62(22.6%)	
Ultrasonic cardiogram (UCG)	135/138(97.8%)	39/40(97.5%)	96/98(98.0%)	1.000

Among the 137 patients who completed lower extremity venous ultrasound, 51 patients had indications of thrombosis, with 12 cases (34.3%) in the non-severe group and 39 cases (38.2%) in the severe group, but the difference was not statistically significant.

VTE risk assessment is a score for venous thromboembolism risk, which helps clinicians identify high-risk patients and assists in the selection of preventive measures to reduce the incidence of VTE. Among the 153 patients who completed VTE risk assessment, 77 patients (50.3%) were classified as low risk (0-2 points), and 75 patients (49.0%) were classified as moderate risk (3-4 points). The non-severe group had a higher proportion of patients classified as low risk (n=34, 64.2%), while the severe group had a higher proportion of patients classified as moderate risk (n=56, 56.0%). There was a statistically significant difference in VTE risk assessment between the non-severe and severe groups (*P*=0.025).

Among the 87 patients who completed abdominal ultrasound, 67 patients had abnormal findings, with 20 cases (71.4%) in the non-severe group and 47 cases (79.7%) in the severe group, but the difference was not statistically significant. Among the 112 patients who completed electrocardiogram (ECG) tests, 34 patients (30.4%) had conduction abnormalities, 23 patients (20.5%) had ST-T changes, and 22 patients (19.6%) had both. There was a significant difference in different ECG manifestations between the non-severe and severe groups (*P*=0.007). Among the 138 patients who completed echocardiography, 135 patients (97.5%) had abnormal findings, with 39 cases (97.5%) in the non-severe group and 96 cases (98.0%) in the severe group, but the data did not show a statistically significant difference between the non-severe and severe groups.

### Univariate and multivariate logistic regression analysis

As shown in **Table 5**, in the univariate logistic regression analysis, aged ≥60 years (*P*=0.015), an elevated systolic blood pressure >140 mmHg (*P*=0.007), a history of smoking (*P*=0.019) and surgery (*P*=0.006) had a higher likelihood of disease progression to severe cases. Among the included laboratory parameters, elevated white blood cell count (*P*=0.004), neutrophil percentage (*P*<0.001), NLR (*P*<0.001), PLR (*P*<0.001), D-dimer (*P*<0.001), fibrinogen (*P*=0.037), IL-6 (*P*<0.001), IL-8 (*P*=0.001), IL-10 (*P*=0.009), CRP (*P*=0.001), PCT (*P*<0.001), catnip (*P*<0.001), BNP (*P*<0.001), CK-MB (*P*=0.003), LDH (*P*<0.001), HBDH (*P*<0.001), uric acid (*P*=0.013) and decreased lymphocyte percentage (*P*<0.001), eosinophil percentage (*P*<0.001), albumin (*P*=0.002), A/G ratio (*P*=0.001), cholinesterase (*P*=0.001), sodium ion (*P*=0.023), calcium ion (*P*<0.001) were closely associated with Omicron variant infection progression to severe cases.

**Table 5:** Univariate and multivariate logistic regression analysis in non-severe and severe groups.

Parameters	Univariate			Multivariate		
	OR	95%CI	*P* value	OR	95%CI	*P* value
Age (<60years vs. ≥60years)	2.615	1.204-5.681	0.015	2.345	0.177-31.129	0.518
WBC count (≤ 9.5×10⁹/L vs. >9.5×10⁹/L)	9.066	2.063-39.840	0.004	14587675	0.000-	0.998
Neutrophil percentage (≤75% vs. >75%)	10.571	4.793-23.316	<0.001	578.899	6.686-50123.367	0.005
Lymphocyte percentage (≥20% vs. <20%)	5.643	2.720-11.705	<0.001	3.560	0.428-29.588	0.240
Neutrophil to lymphocyte ratio (<6.2 vs. ≥6.2)	10.302	4.484-23.668	<0.001	0.138	0.004-4.333	0.260
D-dimer (≤0.55mg/L vs. >0.55mg/L)	6.689	3.087-14.494	<0.001	12.736	1.281-126.603	0.030
Interleukin-6 (≤7pg/mL vs. >7pg/mL)	5.464	2.418-12.347	<0.001	0.463	0.051-4.186	0.493
C-rective protein (≤77mg/L vs. >77mg/L)	4.151	1.760-9.789	0.001	1.105	0.066-18.366	0.945
Troponin I (≤0.023ng/mL vs. >0.023ng/mL)	7.930	2.580-24.376	<0.001	2.594	0.093-72.675	0.575
Creatine kinase-MB(≤5.0ng/mL vs. >5.0ng/mL)	9.270	2.112-40.694	0.003	0.522	0.013-21.406	0.732
Albumin(≥40g/L vs. <40g/L)	11.628	2.444-55.318	0.002	57.758	0.950-3511.918	0.053
Cholinesterase(≥5100U/L vs.<5100U/L)	3.186	1.599-6.347	0.001	0.066	0.006-0.720	0.026
Calcium(≥2.11mmol/L vs.<2.11mmol/L)	13.364	5.912-30.209	<0.001	4.480	0.461-43.525	0.196

**Figure 3:**
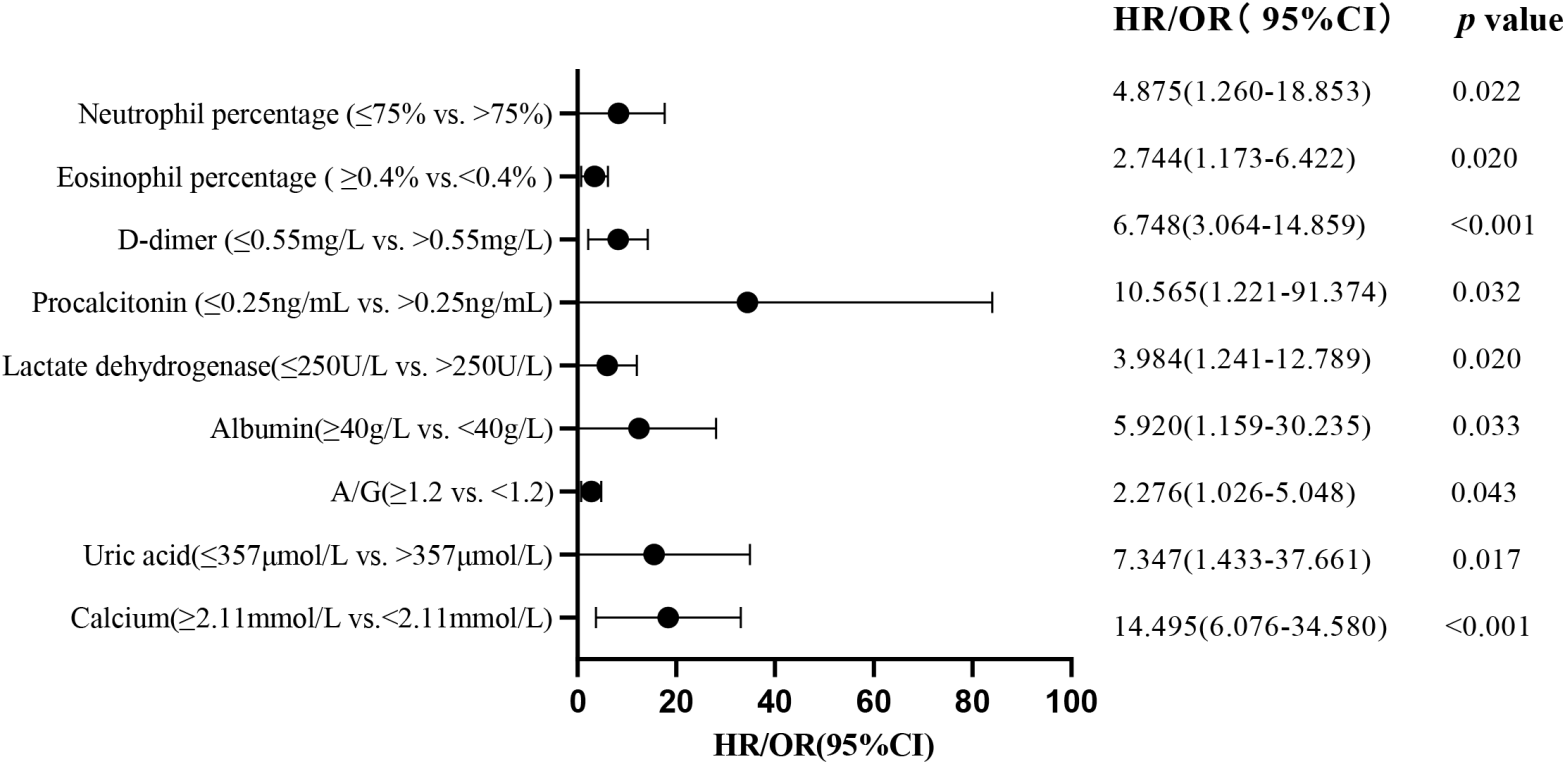
Multivariate regression analysis identifies risk factors for patients with severe Omicron variant infection.

We included 92 patients (23 non-severe and 69 severe) with complete data for all variables in the multivariate logistic regression model (**Table 5**). The included variables were age, white blood cell count, neutrophil percentage, lymphocyte percentage, NLR, D-dimer, IL-6, CRP, catnip, CK-MB, albumin, A/G ratio, cholinesterase, and calcium ion. We found that a neutrophil percentage >75% (OR, 578.899; 95% CI: 6.686-50123.367; *P*=0.005), a D-dimer level >0.55 mg/L (OR, 12.736; 95% CI: 1.281-126.603; *P*=0.030), and a cholinesterase level <5100 U/L (OR, 0.066; 95% CI: 0.006-0.720; *P*=0.026) were significantly associated with an increased likelihood of severe cases (**Figure 3**).

### Multivariate logistic regression model analysis of each system

To further investigate the impact of abnormalities in various organ systems on severe cases of COVID-19, this study included the variables that showed significant differences in the univariate analysis. These variables included blood routine related indicators, inflammation markers, cardiac function markers, liver function markers, renal function markers, and coagulation function markers. These variables were included as independent variables in the logistic regression equation, and whether it is severe or not was used as the dependent variable (**Table 6**). The results showed that in the analysis of blood routine related indicators, an elevated neutrophil percentage (>75%; *P*=0.022) and a decreased eosinophil percentage (<0.4%; *P*=0.020), an elevated PCT (>0.25 ng/mL; *P*=0.032) among the inflammation markers, an elevated LDH (>250 U/L; *P*=0.020) among the cardiac function markers, a decreased albumin (<40 g/L; *P*=0.033) and a decreased A/G ratio (<1.2; *P*=0.043) among the liver function markers, an elevated uric acid (>357 mole/L; *P*=0.017) and a decreased blood calcium ion concentration (<2.11 mmol/L; *P*<0.001) among the renal function markers, and an elevated D-dimer (>0.55 mg/L; *P*<0.001) among the coagulation function markers were all independent risk factors for severe cases in various organ systems. These findings suggest that an elevated neutrophil percentage (>75%), a decreased eosinophil percentage (<0.4%), an elevated PCT (>0.25 ng/mL), an elevated LDH (>250 U/L), a decreased albumin (<40 g/L), a decreased A/G ratio (<1.2), an elevated uric acid (>357 mole/L), a decreased blood calcium ion concentration (<2.11 mmol/L), and an elevated D-dimer (>0.55 mg/L) have higher clinical diagnostic value for severe cases (**Figure 3**).

**Table 6:** Multivariate logistic regression model analysis of multiple systems.

Parameters	Univariate			Multivariate		
	OR	95%CI	*P* value	OR	95%CI	*P* value
Blood routine tests						
WBC count (≤ 9.5×10⁹/L vs. >9.5×10⁹/L)	9.066	2.063-39.840	0.004	8.374	0.984-71.257	0.052
Neutrophil percentage (≤75% vs. >75%)	10.571	4.793-23.316	<0.001	4.875	1.260-18.853	0.022
Lymphocyte percentage (≥20% vs. <20%)	5.643	2.720-11.705	<0.001	1.143	0.381-3.429	0.812
Eosinophil percentage ( ≥0.4% vs.<0.4% )	4.760	2.335-9.704	<0.001	2.744	1.173-6.422	0.020
Neutrophil to lymphocyte ratio (<6.2 vs. ≥6.2)	10.302	4.484-23.668	<0.001	1.725	0.431-6.911	0.441
Coagulation tests						
Prothrombin time (≤12.5s vs. >12.5s)	1.363	0.640-2.900	0.422			
International normalized ratio (≤1.4 vs. >1.4)	9108528	0.000-	0.999			
D-dimer (≤0.55mg/L vs. >0.55mg/L)	6.689	3.087-14.494	<0.001	6.748	3.064-14.859	<0.001
Fibrinogen (≤4.98g/L vs. >4.98g/L)	2.171	1.047-4.505	0.037	2.063	0.927-4.590	0.076
Fibrin degradation products(≤5 vs. >5)	7456037	0.000-	0.998			
Inflammatory indicators						
Interleukin-6 (≤7pg/mL vs. >7pg/mL)	5.464	2.418-12.347	<0.001	1.083	0.200-5.874	0.926
Interleukin-8 (≤20.6pg/mL vs. >20.6pg/mL)	5.689	2.090-15.488	0.001	2.309	0.623-8.563	0.211
Interleukin-10 (≤4.91pg/mL vs. >4.91pg/mL)	3.696	1.381-9.891	0.009	1.850	0.459-7.449	0.387
C-rective protein (≤77mg/L vs. >77mg/L)	4.151	1.760-9.789	0.001	2.230	0.564-8.820	0.253
Procalcitonin (≤0.25ng/mL vs. >0.25ng/mL)	16.638	3.777-73.303	<0.001	10.565	1.221-91.374	0.032
Heart function						
Troponin I (≤0.023ng/mL vs. >0.023ng/mL)	7.930	2.580-24.376	<0.001	1.828	0.453-7.383	0.397
BNP (≤100pg/mL vs. >100pg/mL)	5.385	2.270-12.771	<0.001	3.109	0.988-9.789	0.053
Creatine kinase(≤200U/L vs. >200U/L)	1150749	0---	0.998			
Creatine kinase-MB(≤5.0ng/mL vs. >5.0ng/mL)	9.270	2.112-40.694	0.003	2.056	0.375-11.265	0.406
Lactate dehydrogenase(≤250U/L vs. >250U/L)	6.795	3.151-14-656	<0.001	3.984	1.241-12.789	0.020
Liver function						
Aspartate aminotransferase(≤35U/L vs. >35U/L)	1.568	0.764-3.218	0.220			
Alanine aminotransferase(≤40U/L vs. >40U/L)	0.999	0.462-2.161	0.998			
Albumin(≥40g/L vs. <40g/L)	11.628	2.444-55.318	0.002	5.920	1.159-30.235	0.033
A/G(≥1.2 vs. <1.2)	3.417	1.162-7.243	0.001	2.276	1.026-5.048	0.043
Cholinesterase(≥5100U/L vs.<5100U/L)	3.186	1.599-6.347	0.001	1.872	0.872-4.016	0.108
Total cholesterol (<2.8mmol/L vs. 2.8mmol/L- 3.7mmol/L)	0.600	0.182-1.978	0.401			
Total cholesterol (2.8mmol/L- 3.7mmol/L vs. >3.7mmol/L )	0.766	0.364-1.613	0.483			
High density lipoprotein cholesterol (<1.03mmol/L vs. 1.03mmol/L- 1.55mmol/L)	1.167	0.528-2.578	0.703			
High density lipoprotein cholesterol(1.03mmol/L-1.55mmol/L vs. >1.55mmol/L)	1.658	0.167-16.443	0.666			
Low density lipoprotein cholesterol (<3.37mmol/L vs. ≥ 3.37mmol/L)	0.625	0.179-2.180	0.461			
Kidney function						
Creatinine(≤73μmol/L vs. >73μmol/L)	1.574	0.797-3.110	0.191			
Uric acid(≤357μmol/L vs. >357μmol/L)	6.611	1.487-29.396	0.013	7.347	1.433-37.661	0.017
Sodium(≥137.0mmol/L vs.<137.0mmol/L)	2.294	1.124-4.685	0.023	1.729	0.718-4.162	0.222
Potassium(≥3.5mmol/L vs. <3.5mmol/L)	1.197	0.520-2.759	0.672			
Calcium(≥2.11mmol/L vs.<2.11mmol/L)	13.364	5.912-30.209	<0.001	14.495	6.076-34.580	<0.001

## Discussion

The Omicron variant, a variant of concern, first emerged in the population in November 2021 and was introduced into mainland China on December 9, 2021, gradually becoming the dominant variant in the country (as stated on the official website of the Chinese Center for Disease Control and Prevention). Although many studies suggest that clinical symptoms of Omicron variant infection are milder compared to the Delta variant (Garrett et al., 2022), it is known to have a higher transmissibility than the Delta variant (Chen et al., 2022; Pulliam et al., 2022). Since December 2022, there has been a sharp increase in the number of positive nucleic acid tests for the novel coronavirus in mainland China, with daily new cases exceeding 6 million. The number of hospitalized patients and those in critical condition due to COVID-19 infection has also significantly increased, posing a major public health challenge.

This study shows that the majority of hospitalized patients with the Omicron variant are aged 60 and above. However, other studies on both the Omicron and Delta variants have shown higher infection rates among younger adults (Han et al., 2023). This study also found a significantly higher proportion of elderly patients in the severe group compared to the non-severe group, patients aged 60 or above had a higher likelihood of developing severe illness, which is consistent with other studies on the Omicron variant (Yang et al., 2022). This may be attributed to the overall diminished immune function in the elderly population, suggesting that despite the lower clinical severity of Omicron variant infections compared to the Delta strain (Nyberg et al., 2022; Wolter et al., 2022), the elderly still face a significant threat to their health and lives.

This study found that the proportion of patients with elevated neutrophil percentage was significantly higher in the severe group compared to the non-severe group, and multivariate logistic regression analysis revealed that a neutrophil percentage >75% can serve as a predictor for severe COVID-19. Neutrophils are the main phagocytic cells in the blood and are typically involved in bacterial infections; however, research has also shown that neutrophils can recognize viruses, exert specific effector functions against them, and may contribute to determining disease outcomes (Camp and Jonsson, 2017). The specific mechanisms may involve virus phagocytosis and other immune actions, such as the release of neutrophil extracellular traps (NETs) for viral inactivation (Barr et al., 2018) and cytokine production to restrict viral replication (Lamichhane and Samarasinghe, 2019). Currently, studies have shown that increased neutrophil count can predict adverse outcomes in COVID-19 patients (Wang et al., 2020), with NETs playing an important role, excessive NET formation can lead to a cascade of inflammatory responses, but the precise impact of this inflammatory response on COVID-19 infection is still not well understood and requires further research. There are ongoing studies on the use of NET-targeted approaches for treatment (Cicco et al., 2020), further research will be conducted to explore these aspects in detail.

It has been discovered that SARS-CoV-2 enters cells by binding to angiotensin-converting enzyme 2 (ACE2), which is widely expressed in endothelial cells, SARS-CoV-2 can cause extensive damage to endothelial cells, leading to endothelial dysfunction, which disrupts the balance between coagulation, anticoagulation, and fibrinolysis, resulting in a significant tendency for thrombus formation and activation of the fibrinolysis system, and elevated levels of D-dimer may reflect fibrinolysis (McGonagle et al., 2020). Many studies have shown that the prognosis of COVID-19 can be estimated based on the D-dimer levels upon admission (Cummings et al., 2020; Zhou et al., 2020). This study found that a D-dimer level >0.55 mg/L has a high clinical diagnostic value for severe cases. Early intervention and improvement of coagulation function should be considered for patients with D-dimer levels >0.55mg/L. In our subsequent research, we will further investigate the treatment of anticoagulation and fibrinolysis to find more suitable treatment methods, reduce the occurrence of severe cases, and ultimately reduce mortality rates.

COVID-19 is considered a viral respiratory disease, but numerous studies have found that it also affects other organ systems such as the cardiovascular, liver, kidney, gastrointestinal, and nervous systems (Alqahtani and Schattenberg, 2020; Cheng et al., 2020; Guo et al., 2020; Gupta et al., 2020; Magro et al., 2020). The main manifestations of cardiovascular involvement in COVID-19 include elevated cardiac biomarkers, arrhythmias, acute coronary syndrome, and heart failure (Libby et al., 2018; Arentz et al., 2020; Wang et al., 2020; Ali et al., 2022). Consistently, this study found that compared to the non-severe group, patients in the severe group had more pronounced elevation of cardiac troponin and myocardial enzymes, indicating more significant myocardial injury. Additionally, the occurrence of heart failure, arrhythmias, and myocardial ischemia was higher in the severe group, and these differences were statistically significant. The exact mechanisms underlying COVID-19-related cardiac involvement are still unclear but may involve multiple factors, such as: (1) Direct cellular toxicity to the myocardium through invasion via ACE2, leading to myocarditis (Hoffmann et al., 2020); (2) Cytokine storm and inflammation are also considered mechanisms: inflammatory cytokine levels, such as IL-1β, IL-6, IL-8, and IL-10, were significantly elevated in patients in the severe group, which may be related to cardiovascular involvement; studies have also found that IL-6, as a core cytokine in the cytokine storm, can cause vasculitis, myocarditis, and arrhythmias (Huang et al., 2020), consistent with the findings of this study; (3) Hypoxia and ischemic injury may also be contributing factors (Hoffmann et al., 2020; Xiong et al., 2020), this study also found that the severe group had lower blood oxygen saturation, regardless of whether they received oxygen supplementation or not, supporting this theory. Other studies have suggested that the most common abnormal findings of myocardial injury are abnormal electrocardiograms and elevated cardiac troponin levels, however, relying solely on electrocardiograms and cardiac troponin levels may not accurately diagnose myocardial inflammation, although cardiovascular magnetic resonance (CMR) has a high sensitivity for diagnosing myocarditis, its application is limited in critically ill patients (Ni et al., 2020). This study showed through multivariate analysis that elevated LDH is an independent risk factor for the progression from mild cases to severe cases. LDH is widely present in the cytoplasm and mitochondria of tissue cells, including the liver, heart, and skeletal muscles. It catalyzes the conversion of pyruvate to lactate in the anaerobic oxidation of glucose. During hypoxia, the body primarily obtains energy through anaerobic glucose oxidation, the more pronounced elevation of LDH in severe patients may be related to more severe hypoxia. According to the results of this study, elevated LDH may serve as a potential predictor for severe cases.

According to the “Diagnosis and Treatment Plan for COVID-19 (Trial Version 9)”, COVID-19 patients can also experience liver dysfunction. Studies have found that liver dysfunction caused by SARS-CoV-2 may be the result of multiple factors, including viral replication mediated by ACE-2 in the liver, direct viral-mediated damage, hypoxia or ischemic injury, immune-mediated inflammatory response, drug-induced liver injury, or exacerbation of pre-existing liver disease (Aleem and Shah, 2022; Antinori et al., 2011). The results of this study suggest that abnormalities in liver function indicators are more pronounced in patients with severe disease, manifested primarily by elevated AST and decreased levels of albumin, A/G ratio, and cholinesterase. AST is mainly present in the heart, liver, skeletal muscles, etc., with 80% of it located in the mitochondria of liver cells. Elevated AST indicates mitochondrial damage and reflects severe and persistent liver cell injury. A study conducted in Japan showed that liver function impairment caused by the Delta variant primarily presented as elevated AST, and the increase in AST was more pronounced for the Omicron variant (Suzuki et al., 2022). However, a study conducted in China indicated no significant difference in AST levels between the Omicron variant and the Delta variant (Zhang et al., 2023). The differing results from the two studies mentioned above indicate the need for further collection of more cases to explore the reasons behind these discrepancies. Cholinesterase levels were significantly decreased in the severe group, consistent with other research (Wang et al., 2021). Serum cholinesterase is synthesized in the liver, and reduced enzyme activity often reflects liver damage, but the specific mechanisms underlying liver dysfunction caused by decreased cholinesterase levels are still unclear. As one of the biomarkers of liver dysfunction, decreased cholinesterase levels may also be due to increased capillary wall permeability, enhanced cholinesterase degradation and metabolism, and inhibition by inflammatory mediators (Bahloul et al., 2017). This is consistent with a study conducted by Japanese scholars in early 2020, suggesting that cholinesterase levels may be one of the predictive indicators for the severity and progression of COVID-19 (Kunutsor and Laukkanen, 2021; Nakajima et al., 2021). However, this study suggests that cholinesterase may not be a predictor of disease severity, which may be due to the relatively small sample size of this study or differences in viral variants studied by Japanese scholars. In addition, this study found a significant decrease in albumin levels in the severe group, which is consistent with findings in cases of Delta variant-associated COVID-19 (Chen et al., 2022). Furthermore, studies have found no significant difference in albumin levels between the Omicron variant and the Delta variant, indicating liver damage in both variants without significant differences in the degree of injury. However, this study also found a significant decrease in the A/G ratio in the severe group: on one hand, this may be due to decreased albumin production during liver damage, leading to a lower A/G ratio; on the other hand, globulin is produced by the body’s immune organs, and when viral antigens are present in the body, excessive globulin is produced, leading to an increased denominator in the ratio and causing a decrease in the A/G ratio. More importantly, this study confirms that decreased albumin and A/G ratio can serve as predictive indicators for the progression to severe COVID-19.

Similarly, COVID-19 can also cause renal impairment (Gabarre et al., 2020). This study found that patients in the severe group were more likely to have elevated creatinine levels. The exact mechanisms underlying renal injury caused by SARS-CoV-2 are not yet clear, but studies have suggested that direct viral effects, Renin-Angiotensin-Aldosterone System (RAAS) imbalance, cytokine storms, organ crosstalk, hypercoagulability, complement activation, downregulation of ACE2, microvascular injury, COVID-19-associated prothrombotic state, and other factors may contribute to kidney damage (Gabarre et al., 2020; Zheng et al., 2021; Meng, 2022). The analysis results of this study showed no significant difference in uric acid levels between the non-severe and severe groups, which is contrary to other studies (Huang, 2022). However, in the multivariable analysis including renal impairment, uric acid was found to be a predictive indicator for disease progression to severe cases, which is consistent with other research findings (Dufour et al., 2021), the possible mechanism is that renal tubular damage leads to impaired uric acid reabsorption (Parmaksız and Parmaksız, 2022). Furthermore, it has been observed that patients with hypocalcemia on admission have more severe illness and poorer prognosis (Liu et al., 2020), which is consistent with the findings of this study. In addition, this study identified calcium ion concentration as a predictive factor for disease progression to severe cases. Many factors influence blood calcium metabolism, including parathyroid hormone, calcitonin, and vitamin D. Vitamin D, in particular, works with parathyroid glands to maintain stable blood calcium levels, and studies have found that vitamin D deficiency was positively correlated with the severity of COVID-19 infection (Pereira et al., 2022). Therefore, the specific mechanisms underlying hypocalcemia may involve vitamin D acting as a steroid hormone in immune regulation, changes in intestinal calcium absorption, decreased serum albumin levels, reduced calcium influx, and decreased parathyroid hormone secretion. However, further research is needed to determine which factors play a decisive role. Normal blood calcium levels not only maintain the mineral content of bones but also participate in blood clotting processes, regulate the activity of various enzymes, maintain the integrity and permeability of cell membranes, and influence neuromuscular excitability. This study also found that hypocalcemia can serve as a predictive factor for disease progression to severe cases, which may be related to the impaired coagulation function, reduced myocardial contractility, and abnormal regulation of various enzymes such as protein kinase C, adenylate cyclase, and tyrosine hydroxylase in the presence of hypocalcemia. Further research is needed to elucidate the specific mechanisms involved.

Functional damage to tissues and organs in COVID-19 patients results from various factors. The rapid replication of the novel coronavirus in the human body after infection leads to the overactivation of T cells, immune dysfunction, systemic inflammatory reactions, and dysfunction of the renin-angiotensin system (RAS). This, in turn, results in the overrelease of various proinflammatory cytokines and chemokines, leading to a cytokine storm, with IL-6 being especially prominent. This can lead to damage in various organs, including acute respiratory distress syndrome (ARDS), coagulation disorders, acute renal function impairment, and endothelial dysfunction. The molecular mechanism of novel coronavirus infection and injury is still unclear. The spike protein (S) of SARS-CoV-2 is a major antigen and a target for vaccines. Some people believe that the S protein gene has been evolving, leading to changes in its infectivity and antigenicity (Sharma et al., 2020). There are also studies indicating that SARS-CoV-2 can mutate its spike protein to evade antibodies, and these mutations are already present in some viral mutants transmitted in the population (Weisblum et al., 2020).

The limitations of this study include being a single-center study, a small sample size, incomplete data in some cases, and a lack of follow-up information after transfer or discharge. However, there is currently limited research on the Omicron variant, and this study is at the forefront of research on infections caused by the Omicron variant, as all the Omicron cases in this study were collected based on specific monitoring data from the Chinese Center for Disease Control and Prevention. Currently, the prevalent variant in China is still the Omicron variant, especially since April 21, 2023, when the number of COVID-19 infections in China has been gradually increasing, including a rise in reinfection cases. While the previous Delta variant indiscriminately affected humans, the Omicron variant primarily targets elderly patients. Therefore, understanding the clinical characteristics and prognostic risk factors of Omicron variant infections is of great clinical significance in identifying severe cases among the elderly early on. Based on the results of this study, it is recommended to develop a graded classification and treatment plan for severe COVID-19, considering factors such as age, underlying conditions, vital signs, blood routine tests, inflammatory markers, cardiac, liver, kidney, and coagulation functions, to identify high-risk patients. High-risk individuals include the elderly, those with multiple comorbidities, high neutrophil percentage, low eosinophil percentage, elevated PCT, high LDH, low albumin, low A/G ratio, high uric acid, low blood calcium ion concentration, and elevated D-dimer. Hospitalized patients should receive early and comprehensive treatment, with a multidisciplinary evaluation and intensified intervention to initiate timely intensive care and management, aiming to accelerate patient recovery and provide a basis for the early identification of severe cases in clinical practice.

## Data availability statement

The raw data supporting the conclusions of this article will be made available by the authors, without undue reservation.

## Ethics statement

This study has been approved by the ethics committee of the First Hospital of Hebei Medical University (Ethics Approval No.: S00138). The studies were conducted in accordance with the local legislation and institutional requirements. The participants provided their written informed consent to participate in this study.

## Author contributions

LHW: Conceptualization, Data curation, Formal Analysis, Funding acquisition, Investigation, Writing – original draft. TL: Conceptualization, Formal Analysis, Funding acquisition, Investigation, Software, Validation, Visualization, Writing – original draft. HY: Conceptualization, Formal Analysis, Resources, Writing – original draft. JZ: Data curation, Formal Analysis, Investigation, Methodology, Project administration, Writing – original draft. QS: Conceptualization, Data curation, Investigation, Methodology, Software, Writing – original draft. LW: Data curation, Formal Analysis, Investigation, Methodology, Writing – original draft. XW: Formal Analysis, Investigation, Methodology, Supervision, Writing – original draft. MZ: Funding acquisition, Methodology, Resources, Validation, Writing – original draft. JingW: Formal Analysis, Methodology, Software, Validation, Writing – original draft. JiaW: Conceptualization, Funding acquisition, Supervision, Validation, Visualization, Writing – original draft, Writing – review & editing. WY: Funding acquisition, Supervision, Validation, Visualization, Writing – original draft, Writing – review & editing.
